# Differential MR Delayed Enhancement Patterns of Chronic Myocardial Infarction between Extracellular and Intravascular Contrast Media

**DOI:** 10.1371/journal.pone.0121326

**Published:** 2015-03-27

**Authors:** Jian Wang, Bo Xiang, Hung Yu Lin, Hongyu Liu, Darren Freed, Rakesh C. Arora, Ganghong Tian

**Affiliations:** 1 Department of Vascular Surgery, Union Hospital, Tongji Medical College, Huazhong University of Science and Technology, 1277 Jiefang Street, Wuhan, Hubei, China 430022; 2 National Research Council of Canada, 435 Ellice Avenue, Winnipeg, Manitoba, Canada R3B 1Y6; 3 Department of Physiology, Faculty of Medicine, University of Manitoba, 727 McDermot Avenue, Winnipeg, Manitoba, Canada R3E 3P5; 4 Department of Cardiac Surgery, The First Affiliated Hospital, Harbin Medical University, 23 Youzheng Street, Harbin, Heilongjiang, China 150081; 5 Cardiac Science Program, Institute of Cardiovascular Science, St. Boniface General Hospital, 409 Tache Avenue, Winnipeg, Manitoba, Canada R2H 2A6; University of Cincinnati, College of Medicine, UNITED STATES

## Abstract

**Objectives:**

Because the distribution volume and mechanism of extracellular and intravascular MR contrast media differ considerably, the enhancement pattern of chronic myocardial infarction with extracellular or intravascular media might also be different. This study aims to investigate the differences in MR enhancement patterns of chronic myocardial infarction between extracellular and intravascular contrast media.

**Materials and Methods:**

Twenty pigs with myocardial infarction underwent cine MRI, first pass perfusion MRI and delayed enhancement MRI with extracellular or intravascular media at four weeks after coronary occlusion. Myocardial blood flow (MBF) was determined with microsphere measurement. The infarction histopathological changes were evaluated by hematoxylin and eosin staining and Masson's trichrome method.

**Results:**

Cine MRI revealed the reduced wall thickening in chronic infarction compared with normal myocardium. Moreover, significant wall thinning in chronic infarction was observed in cine MRI. Peak first-pass signal intensity didn’t significantly differ between chronic infarction and normal myocardium no matter what kinds of contrast media. At the following delayed enhancement phase, extracellular media-enhanced signal intensity was significantly higher in chronic infarction than in normal myocardium. Conversely, intravascular media-enhanced signal intensity was almost equivalent among chronic infarction and normal myocardium. At four weeks after infarction, MBF in chronic infarction approached to that in normal myocardium. Large thick-walled vessels were detected at peri-infarction zones. The cardiomyocytes were replaced by scar tissue consisting of dilated blood vessels and discrete fibers of collagen.

**Conclusions:**

Chronic infarction was characterized by the significantly reduced wall thickening and the definite wall thinning. First-pass myocardial perfusion defect was not detected in chronic infarction with two media due to the significantly recovered MBF and well-developed collateral vessels. Infarction remodeling enlarged the extracellular compartment, which was available for extracellular media but not accessible to intravascular media. Extracellular media identified chronic infarction as the hyper-enhancement; nonetheless, intravascular media didn’t provide delayed enhancement.

## Introduction

Comprehensive MRI allows for a simultaneous state-of-the-art analysis of wall thickness and thickening, myocardial perfusion and the location and size of infarction [1]. Cine MRI is accepted as a valuable noninvasive method of assessing wall thickness and wall thickening [2–4]. End-diastolic wall thickness and resting wall motion grade can predict recovery of regional dysfunction after revascularization [5]. Ischemic myocardial necrosis is the main reason for the reduced wall thickening post-infarction. The thin-walled collagenous scar eventually takes over the necrotic myocardium. MRI scar of patients was defined as diastolic wall thickness 2.5 standard deviation below corresponding normal values or systolic wall thickening less than or equal to 1 mm [4]. MRI study of rat myocardial infarction (MI) has demonstrated that wall thinning occurs and evolves since 4 weeks post-infarction [6]. This study investigated the regional wall thickening and wall thinning at 4 weeks post-infarction in a pig animal model.

The first-pass contrast kinetic relies mainly on intravascular volume and blood flow velocity. The coronary vasculature in jeopardized region can be impaired or even obstructed due to ischemia and reperfusion injury after coronary occlusion. Diminished myocardial blood flow (MBF) limits the contrast transit through the jeopardized regions, resulting in a differential enhancement between normal and infracted regions. Microvascular obstruction, typically present in acute reperfused MI, is visualized as a hypo-enhancement on first-pass perfusion MRI [7, 8]. This is mainly due to endothelial swelling and obstruction of the capillaries by blood cells and debris [9, 10]. Moreover, first-pass perfusion MRI has been demonstrated to depict 5-day-old occlusive infarction as a perfusion defect [11]. The pathological changes of acute and chronic MI differ considerably; whether first-pass perfusion MRI identifies the chronic MI as a perfusion abnormality is not well understood.

The delayed enhancement MR pattern relies principally on the diffusion properties of contrast media, the vascular permeability, and the enlarged interstitial space. Extracellular media of small molecular weight distribute freely in the interstitial compartment. The disruption of myocyte membrane after prolonged ischemia enlarges the interstitial space accessible to extracellular media and increases its distribution coefficients in the acute MI [12, 13]. Conversely, intravascular media are confined to the intravascular space in normal myocardium; however, damaged or hyper-permeable microvessels of infarcted tissues allow intravascular media to leak into interstitial spaces [14, 15]. It is unknown whether vascular remodeling after MI results in the hyper-permeability of residual vessels in scar tissue. Therefore, whether intravascular media produce the delayed enhancement of chronic MI remains poorly understood.

Gadolinium diethylenetriamine pentaacetic acid (Gd-DTPA) has a molecular weight of 938 D, and a 0.9-nm diameter, which allows its distribution in the extracellular compartment [16]. P792 belongs to new macromolecular gadolinium-based macromolecules. P792 has a molecular weight of 6473 D, and a diameter of 5nm, which restricts fast diffusion out of normal microvessel but is small enough to be filtered by the kidney [17]. Because the distribution volume and mechanism of extracellular and intravascular MR contrast media differ considerably, the enhancement pattern with extracellular and intravascular media might also be different.

Therefore, the purpose of this study was to characterize chronic MI in comprehensive MRI comprising cine, first-pass and delayed enhancement MR imaging and was to clarify the differences in MR enhancement pattern of chronic MI between extracellular and intravascular contrast media.

## Materials and Methods

### Ethics Statement

This study was conducted under an approval by the Institional Review Board and Animal Care Committee of National Research Council of Canada and Huazhong University of Science and Technology (Permit Number: 2008037).

### Animal experimental preparation

Twenty domestic pigs weighing 20–25 kg were intubated and ventilated with gas anesthesia comprising 1%–2% isoflurance in a mixture of oxygen and nitrous oxide. The left thoracotomy was performed in the fourth left intercostals space and the pericardium was opened to expose the heart. MI was created by ligating the first and second diagonal branches of left anterior descending coronary artery. The chests were closed and the animals were allowed to recover for 4 weeks for MRI examination.

### Experimental protocol

Twenty pigs were studied 4 weeks after coronary occlusion. Animals were anesthetized, intubated, transported to the MRI facility. All pigs were divided into two groups (n = 10, per group). In Group I, pigs initially received the bolus administration of 0.1 mmol/kg Gd-DTPA (Magnevist, Berlex Canada Inc, Pointe-Claire, Quebec, Canada); 80 minutes later, another bolus of 0.026 mmol/kg P792 (Vistarem, Guerbet Group, Paris, France) was administrated intravenously. In Group II, pigs initially underwent the bolus injection of 0.026 mmol/kg P792; after 80 minutes, another bolus of 0.1 mmol/kg Gd-DTPA was administrated intravenously. Our study demonstrated that 80 minutes was enough to remove most contrast media from blood by kidney. The MR protocol comprised cine MRI, first-pass perfusion MRI and delayed enhancement MRI. The cine MRI was conducted prior to contrast administration. Each pig possessed two sets of delayed enhancement and first-pass MR images with Gd-DTPA and P792, respectively. The second set of MR images were acquired in the same orientation with the first set of MR images. After MRI examination, all hearts were removed and sectioned from base to apex. Alternating sections were used for histochemical staining, with the opposing side used for colored microsphere analysis.

### Magnetic resonance imaging

All pigs were imaged in the lateral decubitus position using a state-of-the-art 3.0T MR scanner (Magneton Symphony Vision, Siemens AG, Erlangen, Germany). A four-element (two anterior and two posterior) cardiac array receiver surface coil was utilized for signal reception. ECG electrodes were attached to trigger MR acquisition. Cine images were acquired using true fast imaging with steady-state precession (TrueFISP). Breath-hold was performed at tidal inspiration. Contiguous 14 slices of 6.5mm thickness were acquired to cover the whole left ventricle (LV) in short-axis view. The parameters of FLASH were as follows: Field of view: 300, Repetition time: 29.22ms, Echo time: 2.4ms, Flip Angle: 12°, and acquisition matrix: 256 × 224. The following parameters were used for TrueFISP: Field of view: 310, Repetition time: 4.2ms, Echo time: 1.5ms, Flip Angles: 60°, and acquisition matrix: 224 × 224. First-pass perfusion MR images were performed using a multislice saturation prepulse gradient-recalled echo (SR-prepared GRE) sequence in short axis planes. The sequence allows us to achieve the entire stack of images every two RR interval. The following imaging parameters were used: Field of view: 360, Repetition time: 150.97ms, Echo time: 2.17ms, Inversion time: 90ms, Slice thickness: 5mm, Flip Angle: 12°, acquisition matrix: 111 × 192. A single dose of contrast medium was infused intravenously at a flow rate of 3ml/s. The bolus administration was performed 3 seconds after onset of fast sequence, allowing the acquisition of at least three pre contrast images during the period of 60 second. The delayed enhancement MR images were performed in short-axis planes with the inversion recovery gradient-recalled echo (IR-prepared GRE) sequence. The delayed images were acquired 4 minutes after contrast administration and repeated for 6 points for 24 minutes. The following parameters were used: Flip angle: 20°, Slice thickness: 5mm, Repetition time: 564ms, Echo time: 1.56ms, and acquisition matrix: 123 × 256. Inversion time varied from 200 to 300 msec dependent on the myocardium nulling. All delayed images were acquired from apex to base in the end diastole of LV.

### Colored microsphere technique

After MRI examination, approximately 5–8×10^6^ nonradioactive red-colored microspheres were injected into the peripheral vein. Arterial reference samples were withdrawn simultaneously from carotid artery at a constant rate of 5 ml/min for 3 min, starting 1 min before injection. The hearts were then removed from the animals under full-depth anesthesia. Myocardial samples were collected from the LV wall in a pie-shape. The blood and myocardial samples were digested. The numbers of microspheres in the samples were counted using a spectrophotometer at a wavelength of 536 nm. MBF was then calculated based on the microsphere count in tissue and blood samples as well as the speed of blood collection.

### Histological examination

The slices were firstly stained with 2% triphenyltetrazolium chloride (TTC) and photographed under room temperature to localize the MI. The slices were then separated into infarction scar and normal myocardium. The samples were fixed in 10% formalin, embedded in paraffin, and sectioned at 8μm thickness. Sections were stained with hematoxylin and eosin. Masson’s trichrome stain was used to define scarred myocardium.

### MRI data analysis

MRI data analysis was performed in using freely available software (Segment, Version 1.8R0438, http://segment.heiberg.se). Epicardial and endocardial borders were automatically outlined in serial short-axis cine MR images. Papillary muscles and trabeculation were excluded from the myocardium. We used 6 segmental model, in which mid-ventricular slices were divided into anterio-septal, anterior, anterio-lateral, interio-lateral, interior, interio-septal wall. The anterio-lateral wall of LV was defined as the MI due to the hyper-enhancement by Gd-DTPA and positive staining by TTC. Normal myocardium included anterior, inferior and interio-lateral wall without hyper-enhancement and TTC positive staining. Measurements of wall thickness (WT) were obtained in three contiguous mid-ventricular short-axis images. Wall thickening was calculated with the following equation: wall thickening = (WT_end-systole_—WT_end-diastole_) × 100/WT_end-diastole_. Contrast enhanced signal intensities were measured in normal myocardium, chronic MI and LV chamber blood. These regions were defined on the basis of TTC stained picture and wall motion. The MI region was explained as the reduced wall motion and TTC positive staining in the dominant territory of ligated coronary. Normal Myocardium referred to the LV region opposite to MI. Delayed enhancement signal intensities were qualified and were expressed as the percent increase compared with pre-contrast baseline value. Normalized time intensity curves were sampled at six time points to describe the time course of delayed enhancement beyond contrast first pass. The locations of these regions (normal myocardium, chronic MI and LV chamber blood) were then transferred to first-pass perfusion MR images. Care was taken to define the regions of interest several pixels from epicardial and endocardial surfaces to avoid partial volume effects. Myocardial time intensity curves during contrast transit were then generated within these regions. Time intensity curves were normalized by expression of all signal intensities as a percentage of the fully relaxed intensities.

### Statistical analysis

All data was presented as the mean ± standard deviation. Signal intensities between normal and infarcted myocardium at specific time points were compared by Bonferroni’s t test. The changes over time of signal intensity obtained from infarcted myocardium were compared with the patterns obtained from normal myocardium by repeated measures analysis of variance (ANOVA). Wall thickness, systolic thickening and MBF between normal and infracted myocardium were compared with a paired *t* test. Values of *P* < 0.05 were deemed significant.

## Results

### Wall thickening in chronic MI by cine MR images

**Fig 1:**
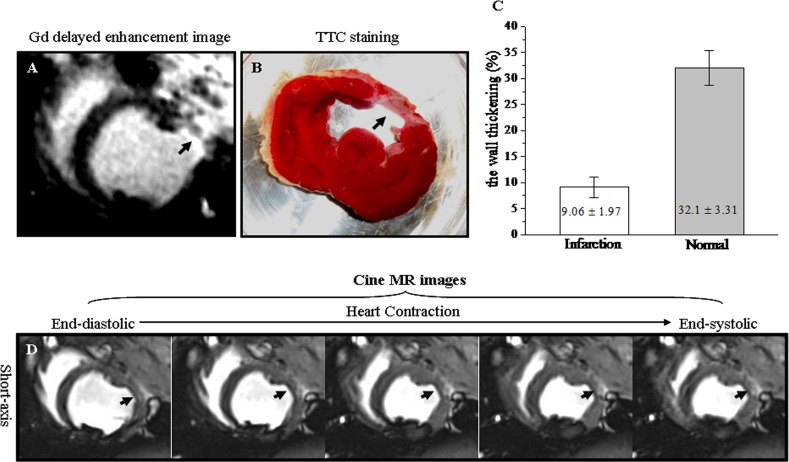
The wall thickening in chronic infarction by cine MR images. A. Gd-DTPA delayed enhancement image. B. Corresponding TTC staining picture. C. Wall thickening in chronic MI and normal myocardium. D. Corresponding left ventricular short-axis cine images obtained from end-diastole to end-systole during the cardiac cycle. The chronic anterio-lateral wall infarction was hyper-enhanced in Gd-DTPA delayed enhanced image (Arrowhead, Fig. 1A) and was confirmed in TTC staining picture (Arrowhead, Fig. 1B). The wall thickening was significantly lower in chronic infarction than in normal myocardium (Fig. 1C). Wall thickness in the anterio-lateral hyper-enhanced chronic infarction remained unchanged with heart contraction (Arrowheads, Fig. 1D).

Gd-DTPA delayed enhancement MR image and TTC staining picture demonstrated that MI was located in the anterio-lateral wall of LV (Arrowheads, Fig. 1A and 1B). The wall thickening in chronic MI (9.06% ± 1.97%) was significantly reduced compared with normal myocardium (32.10% ± 3.31%) (Fig. 1C). The corresponding cine MR images in LV short-axis view during the whole cardiac cycle were illustrated In Fig. 1D. The anterio-lateral wall thickness remained unchanged from end-diastole to end-systole with heart contraction (Arrowheads, Fig. 1D). These demonstrated that 4-week chronic MI showed the significantly depressed wall thickening (S1 Video).

### Wall thickness in chronic MI by cine MR images

**Fig 2:**
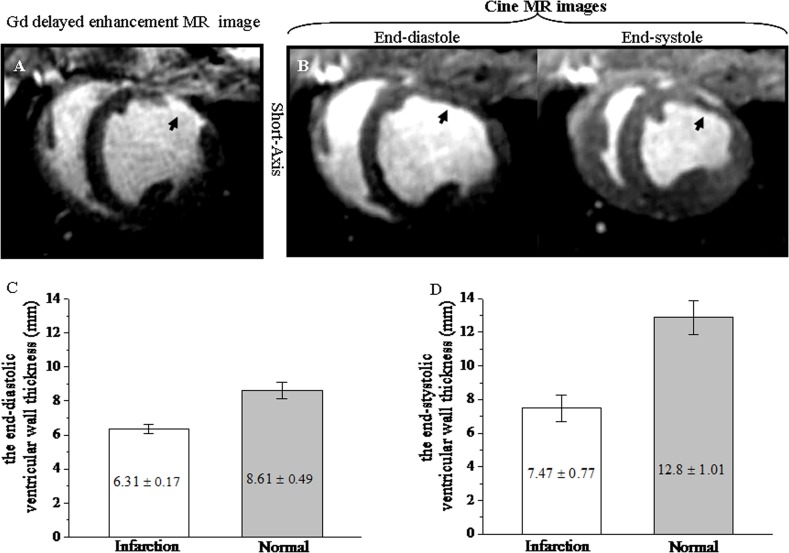
The wall thickness in chronic infarction by cine MR images. A. Left ventricular short-axis Gd-DTPA delayed enhancement image. B. Corresponding left ventricular short-axis cine images obtained from end-diastole and end-systole. C. End-diastolic wall thickness in chronic infarction and normal myocardium. D. End-systolic wall thickness in chronic infarction and normal myocardium. The chronic anterior-lateral wall infarction was identified as the hyper-enhanced region in Gd-DTPA delayed enhanced image (Arrowhead, Fig. 2A). The anterio-lateral wall thinning was significant in end-diastolic and end-systolic cine images (Arrowheads, Fig. 2B). The end-diastolic and end-systolic wall thicknesses were significantly lower in chronic infarction than in normal myocardium (Fig. 2C and 2D).

The chronic anterio-lateral wall infarction was identified as the hyper-enhancement in Gd-DTPA delayed enhancement image (Arrowhead, Fig. 2A). The corresponding cine MR images from end-systole and end-diastole in LV short axis views were illustrated in Fig. 2B. The significant wall thinning was observed in anterio-lateral chronic MI (Arrowheads, Fig. 2B). The end-diastolic wall thickness was markedly lower in chronic MI (6.31 ± 0.17mm) than in normal myocardium (8.61 ± 0.49) (Fig. 2C). Likewise, the end-systolic wall thickness was dramatically smaller in chronic MI (7.47 ± 0.77) than in normal myocardium (12.8 ± 1.01) (Fig. 2D). These suggested that chronic MI developed a significant wall thinning at 4 weeks post-infarction (S2 Video).

### First-pass perfusion MR images and time intensity curves of Gd-DTPA

**Fig 3:**
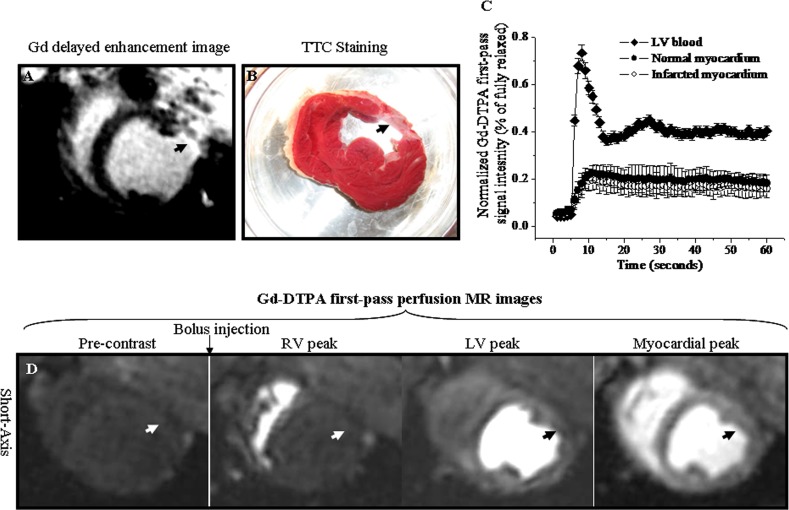
First-pass perfusion MR images and time intensity curves of Gd-DTPA. A. Gd-DTPA delayed enhancement image. B. Corresponding TTC staining picture. C. Time intensity curves obtained from the first passage of Gd-DTPA. D. Corresponding Gd-DTPA first-pass perfusion images. The chronic anterior-lateral wall infarction was hyper-enhanced in Gd-DTPA delayed enhanced image (Arrowhead, Fig. 3A) and was confirmed in TTC staining picture (Arrowhead, Fig. 3B). The bolus of Gd-DTPA resulted in a rapid and equivalent signal increase in chronic infarction and normal myocardium (Fig. 3C). The chronic infarction did not display perfusion defect with less enhancement relative to normal myocardium (Arrowheads, Fig. 3D).

The Gd-DTPA delayed enhancement image and TTC staining picture served as the MI localization (Arrowheads, Fig. 3A and 3B). Time intensity curves obtained during Gd-DTPA first passage were illustrated in Fig. 3C. LV chamber blood signal was significantly enhanced at the effect of the bolus Gd-DTPA from 4.28% ± 0.2% to 73.19% ± 3.49% of fully relaxed intensity (Fig. 3C). The bolus injection of Gd-DTPA caused a similar signal increase of normal myocardium (22.5% ± 3.19%) and chronic MI (19.1% ± 3.65% of fully relax) (Fig. 3C). The corresponding first-pass perfusion MR images with bolus Gd-DTPA were illustrated in Fig. 3D. The anterio-lateral chronic MI didn’t display the perfusion defect with fewer enhancements during Gd-DTPA first passage (Arrowheads, Fig. 3D). These suggested that chronic MI was indistinguishable from normal myocardium during Gd-DTPA first passage (S3 Video).

### First-pass perfusion MR images and time intensity curves of P792

**Fig 4:**
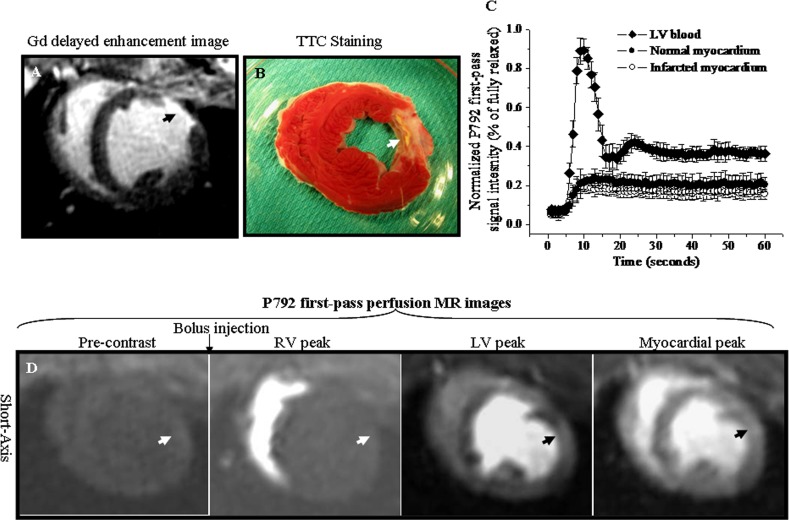
First-pass perfusion MR images and time intensity curves of P792. A. Gd-DTPA delayed enhancement image. B. Corresponding TTC staining picture. C. Time intensity curves obtained from the first passage of P792. D. Corresponding P792 first-pass perfusion images. The chronic anterior-lateral wall infarction was hyper-enhanced in Gd-DTPA delayed enhanced image (Arrowhead, Fig. 4A) and was confirmed in TTC staining picture (Arrowhead, Fig. 4B). The bolus of P792 caused a rapid and equivalent signal increase in chronic infarction and normal myocardium (Fig. 4C). The chronic infarction did not show perfusion abnormalities with fewer enhancements relative to normal myocardium (Arrowheads, Fig. 4D).

The Gd-DTPA delayed enhancement image and TTC staining picture highlighted the position of chronic MI (Arrowheads, Fig. 4A and 4B). Time intensity curves obtained from P792 first passage were illustrated in Fig. 4C. The bolus administration of P792 resulted in a rapid signal increase of LV chamber blood from 7.77% ± 1.12% to 88.8% ± 6.73% of fully relax (Fig. 4C). Transit of P792 caused the uniform and equal signal increase between chronic MI and normal myocardium. The peak signal intensities were 21.8% ± 3.65% and 18.7% ± 2.84% of fully relax in normal myocardium and chronic MI, respectively (Fig. 4C). The corresponding first-pass MR images with bolus P792 were illustrated Fig. 4D. The perfusion abnormalities with fewer enhancements were not observed in anterio-lateral chronic MI (Fig. 4D). These suggested that P792 first-pass MR image could not identify chronic MI as the perfusion deficiency (S4 Video).

### Gd-DTPA delayed enhancement MR images and signal intensities

**Fig 5:**
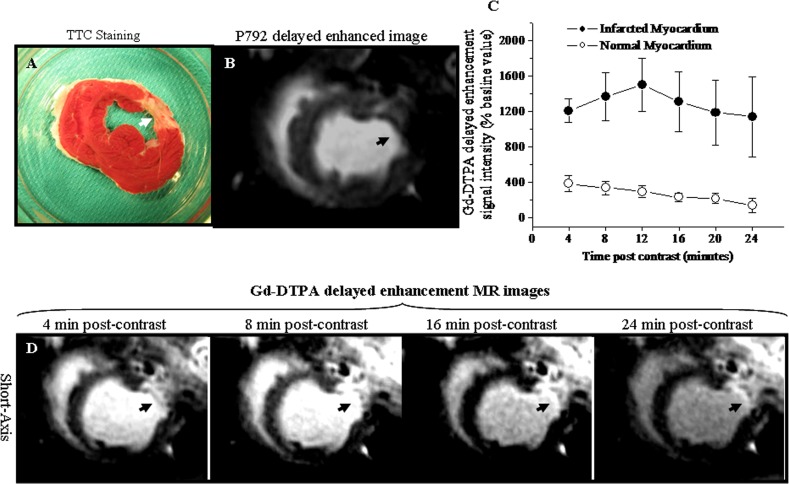
Gd-DTPA delayed enhancement MR images and signal intensities. A. TTC staining picture. B. Corresponding P792 delayed enhancement image. C. Gd-DTPA enhanced signal intensities at various time points post-injection. D. Corresponding left ventricular short-axis Gd-DTPA delayed enhancement images obtained at different time points post-injection. The chronic anterior-lateral wall infarction was confirmed in TTC staining picture (Arrowhead, Fig. 5A). The differential delayed enhancement pattern was not observed in the corresponding P792 delayed enhancement image (Arrowhead, Fig. 5B). However, the Gd-DTPA enhanced signal intensity was dramatically higher in chronic infarction than in normal myocardium (Fig. 5C). Thus, the chronic infarction was identified as the hyper-enhancement in the corresponding Gd-DTPA delayed enhancement images (Arrowheads, Fig. 5D).

TTC staining picture indicated that MI was located in the anterio-lateral wall of LV (Arrowhead, Fig. 5A). The corresponding P792 delayed enhancement MR image seemed not to distinguish chronic MI as hyper-enhancement (Arrowhead, Fig. 5B). However, Gd-DTPA delayed enhancement signal intensity of chronic MI ranged from 1210.7% ± 131.6% to 1502.7 ± 303.1% of the baseline value for different time points post-administration and was significantly higher than observed in normal myocardium, ranging from 387.8% ± 91.6% to 143.8% ± 79.8% of baseline value (Fig. 5C). Thus, anterio-lateral chronic MI exhibited delayed hyper-enhancement in the corresponding Gd-DTPA delayed enhancement MR images (Arrowheads, Fig. 5D). These suggested that chronic MI was distinguishable from normal myocardium in Gd-DTPA delayed enhancement MR images.

### P792 delayed enhancement MR images and signal intensities

**Fig 6:**
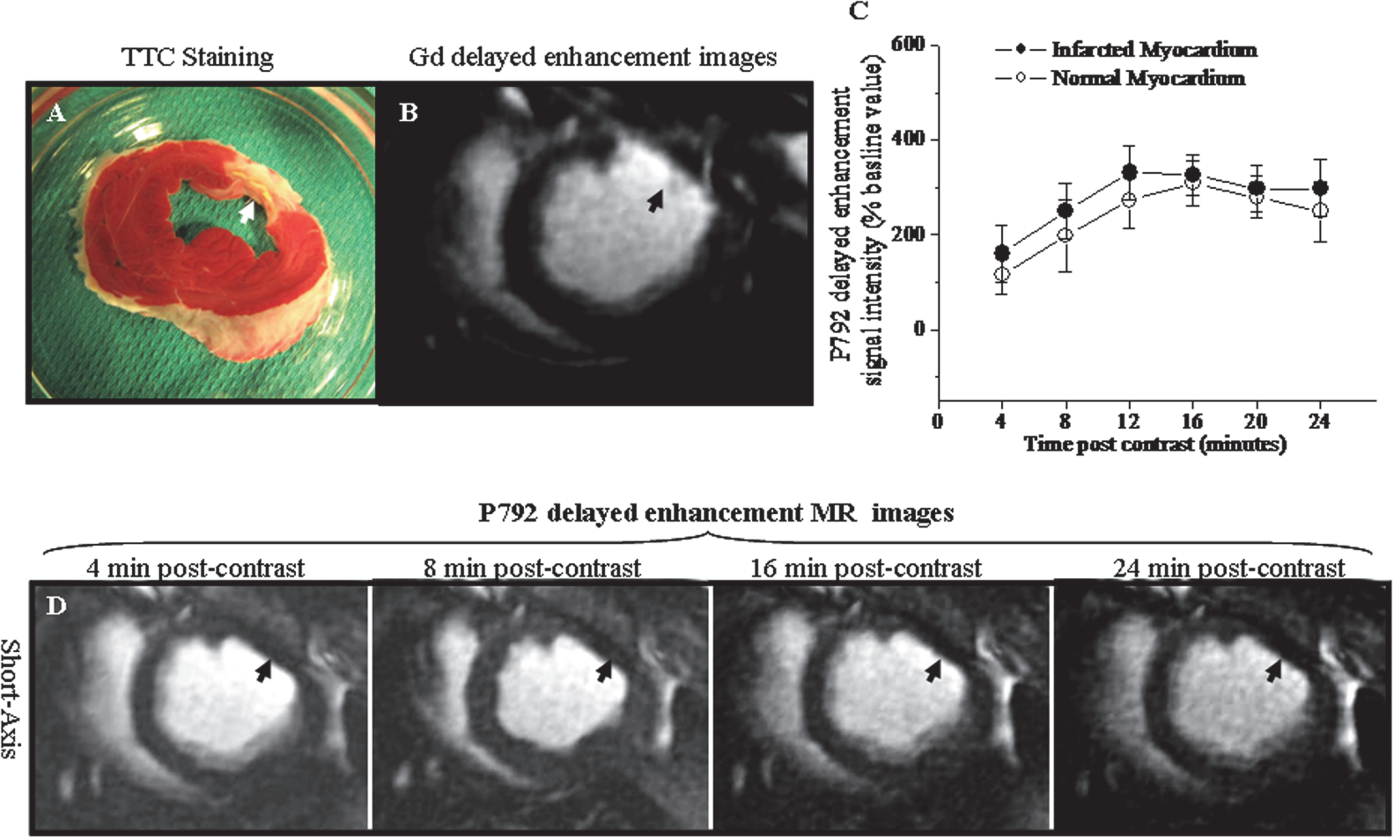
P792 delayed enhancement MR images and signal intensities. A. TTC staining picture. B. Corresponding Gd-DTPA delayed enhancement image. C. P792 enhanced signal intensities at various time points post-injection. D. Corresponding left ventricular short-axis P792 delayed enhancement images obtained at different time points post injection. The chronic anterio-lateral wall infarction was confirmed in TTC staining picture (Arrowhead, Fig. 6A). The chronic MI displayed the delayed hyper-enhancement in Gd-DTPA delayed enhancement images (Arrowhead, Fig. 6B). However, the P792 enhanced signal intensity in chronic infarction did not differ from that in normal myocardium (Fig. 6C). Thus, the P792 did not produce differentiated hyper-enhancement of chronic MI (Arrowheads, Fig. 6D).

TTC staining picture indicated that MI was located in the anterio-lateral wall of LV (Arrowhead, Fig. 5A). The corresponding Gd-DTPA delayed enhancement MR image distinguished chronic MI as hyper-enhancement (Arrowhead, Fig. 6B). However, P792 delayed enhancement signal intensity of chronic MI ranged from 160.3% ± 60.9% to 331.2 ± 55.4% of the baseline value for different time points post-administration and was not statistically different from that observed in normal myocardium, ranging from 116.1% ± 40.8% to 309.3% ± 48.0% of baseline value (Fig. 6C). Thus, the corresponding P792 delayed enhancement MR images didn’t provide differential enhancement between anterio-lateral chronic MI and normal myocardium (Arrowheads, Fig. 6D). These suggested P792 delayed enhancement MR images exhibited the homogeneous enhancement between chronic MI and normal myocardium.

### Histology and myocardial blood flow

**Fig 7:**
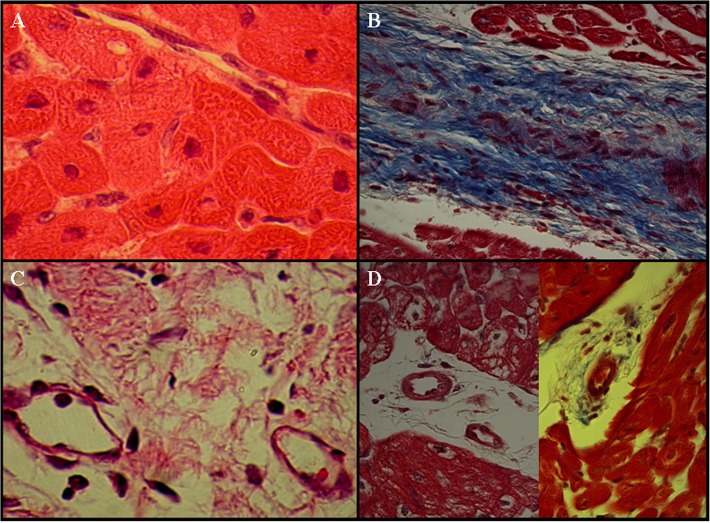
Histopathology of chronic myocardial infarction at four weeks after myocardial infarction. A. Cardiomyocytes were the major structural elements in normal myocardium. B. Scar tissue was characterized by the discrete collagen fibers and loss of cellularity. Collagen fibers were stained blue using Masson’s trichrome staining (Arrowhead, Fig. 7B). C. Dilated remodeled vessels were frequently observed in the territory of the infarction (Arrowheads, Fig. 7C). D. Large thick-walled blood vessels were apparent at the peri-infarction zones (Arrowheads, Fig. 7D).

The major structural elements were the intact cardiomyocytes and there existed relatively small extracellular space accessible to contrast media in normal myocardium (Fig. 7A). In chronic MI, the extracellular space enlargement was mostly attributed to the sparse collagen fibrous deposition (Arrowhead, Fig. 7B) and loss of cardiomyocytes. Residual dilated vessels were detected with infarction (Arrowheads, Fig. 7C), and large thick-walled blood vessels were manifest at the peri-infarction zone (Arrowheads, Fig. 7D). Regional MBF in infarction scar (0.91 ± 0.11 ml/min/g) approximately recovered to close-normal level (1.06 ± 0.08 ml/min/g). Significantly restored MBF was able to deliver contrast media into infarction scar through well-developed collateral vessels. The vascular permeability of scar tissue allowed only extracellular not intravascular contrast media to leak into the enlarged extracellular compartment, producing the differential delayed enhancement MR patterns between two kinds of media.

## Discussion

Cine MR imaging using SSFP relies mainly on T_1_ and T_2_ contrast to provide high contrast between intracavity blood and the endocardium without contrast media, and allows accurate delineation of endocardium and epicardium, therefore permitting an exact determination of regional wall thickness and wall thickening. In acute stage of permanent coronary occlusion, the myofibroblast dramatically proliferates and migrates into the infarct zone [18, 19]. As infarction remodeling, the myofibroblast gradually deposits into a collagenous network, which completely replaces the necrotic myocardium until 4 week post-infarction [18, 19]. The contractile features of myofibroblasts contribute to the progressive resorption of infarction and thinning of infracted ventricular wall [20]. In this study, the chronic MI showed the negligible wall thickening and significant wall thinning at 4 weeks following infarction. Consistent with our founding is a report by Matthias, who found the significant wall thinning in rats models from 4 weeks to 16 weeks after infarction [21].

The first-pass perfusion MRI generally relies on a T_1_-weighted sequence to create images that enhance with the passage of contrast media through the circulation and into coronary bed. In myocardium supplied by a stenotic or occluded coronary artery, impaired MBF causes a slower arrival and lower contrast concentration relative to normal myocardium; and myocardium with perfusion abnormality appears as a black patch with fewer enhancements. Signal intensity time curves of the first-pass MR perfusion images are used to assess regional variations in MBF [22]. After the rapid bolus of intravascular or extracellular media, a rapid and dramatic signal increase was observed in both normal and 4 week-old scarred myocardium due to wash-in of contrast media. The fact that signal increase in chronic MI was almost equivalent to that in normal myocardium during the wash-in of both agents was of considerable importance, because it strongly indicated that occurrence and development of collateral circulation resulted in a significant restoration of MBF in chronic MI. Histological examination of scar demonstrated that the absence of first-pass MR perfusion defect was primarily attributed to the significantly restored MBF and well-developed collateral vessels. Investigation in swine models found that a marked collateral network had been observed to cause regional flow to increase 14-fold at 3–4 week post-occlusion relative to initial occlusion [23] and approached normal levels within 3–7 weeks post-occlusion [24]. More significantly, the vast majority of capillaries disappeared within 3 weeks after infarction, whereas many dilated vessels containing at least 1 layer of smooth muscle cells persisted in scarred myocardium [25]. The average area occupied by vessels did not change between scarred and normal myocardium [26].

Four weeks after coronary occlusion, two differential delayed enhancement MR patterns were observed with intravascular and extracellular contrast media. Chronic MI was identified as a hyper-enhancement in extracellular agent delayed enhancement images. This was in agreement with prior studies which demonstrated that chronic MI was commonly observed as a region of hyper-enhancement in conjunction with extracellular media [27, 28]. Moreover, there was evidence to suggest that regional elevations in Gd-DTPA concentration are exclusively associated with chronic MI showing delayed hyper-enhancement [29]. Microscopic examinations of scar tissue showed thick-walled large blood vessels and massive microvessels [18, 19]. Histological staining section clearly indicated that scar tissue was deficient in cellularity and mainly comprised discrete matrix deposition [18, 19]. Collateral circulation formation and residual vessel effectively delivered contrast media into scarred myocardium [30], and discrete collagen fiber meshwork and loss of cellularity enlarged extracellular space accessible to Gd-DTPA, eventually resulting in the delayed hyper-enhanced scar [30, 31]. Unlike extracellular media, intravascular media did not produce the differential enhancement between chronic MI and normal myocardium. This strongly indicated that vascular integrity was intact in scar tissue. Intravascular media are still confined within intravascular space in chronic MI. Although there is a relatively enlarged interstitial space in chronic MI due to loss of cardiomyocytes and discrete matrix deposition, the intact microvascular permeability is not able to permit the leakage of intravascular media into the enlarged interstitial space and create an increased distribution volume.

## Conclusions

The chronic MI displayed the negligible wall systolic thickening and significant wall thinning in cine MRI. The bolus of extracellular or intravenous media resulted in a dramatic and uniform signal increase between chronic infarction and normal myocardium. The absence of first-pass MR perfusion defect was mostly due to the significantly restored collateral circulation in chronic MI. The vascular integrity was intact in chronic scar infarction and did not allow intravascular media to leak into the extracellular space. Therefore, the chronic MI was identified as an area of the delayed hyper-enhancement with extracellular media; nonetheless, intravascular media did not enhance chronic MI.

## Supporting Information

S1 Video Left ventricular short-axis cine MR images in chronic infarction. Four weeks after myocardial infarction, the wall thickness in chronic anterio-lateral wall myocardial infarction was almost unchanged during the whole cardiac cycle. The wall thickening in chronic infarction was significantly depressed to approximately one-third of the value of normal myocardium.
(AVI)

S2 Video Left ventricular short-axis MR images in chronic infarction. The significant decrease in wall thickness of the anterio-lateral infarction scar was detected at four weeks post-infarction. There was significant thinning of the chronic anterio-lateral scar infarction.
(AVI)

S3 Video Gd-DTPA enhanced first-pass MR myocardial perfusion images. Four weeks after myocardial infarction, the bolus injection of Gd-DTPA caused a rapid and uniform signal increase in chronic infarction and normal myocardium during first-pass period. Therefore, the first-pass perfusion MRI could not distinguished chronic anterio-lateral wall infarction and normal myocardial.
(AVI)

S4 Video P792 enhanced first-pass MR myocardial perfusion images. Four weeks after myocardial infarction, the bolus injection of P792 caused a rapid and equivalent signal increase in chronic infarction and normal myocardium during first-pass period. Thus, the chronic anterio-lateral wall infarction did not show the perfusion defect with fewer enhancements compared with normal myocardium.
(AVI)
